# Reinterpretation of Health Information in the Context of an Emerging Infectious Disease: A Digital Focus Group Study

**DOI:** 10.2196/39312

**Published:** 2022-11-22

**Authors:** Rayner Kay Jin Tan, Jane Mingjie Lim, Pearlyn Hui Min Neo, Suan Ee Ong

**Affiliations:** 1 University of North Carolina Project-China Guangzhou China; 2 Saw Swee Hock School of Public Health National University of Singapore and National University Health System Singapore Singapore; 3 Dermatology Hospital of Southern Medical University Guangzhou China; 4 Research for Impact Singapore Singapore

**Keywords:** health communication, infodemic, SARS-CoV-2, coronavirus, Singapore, WhatsApp, COVID-19, health information, misinformation, mobile health, smartphone, information quality, online health information

## Abstract

**Background:**

Misinformation related to the COVID-19 pandemic has accelerated global public concern and panic. The glut of information, or “infodemic,” has caused concern for authorities due to its negative impacts on COVID-19 prevention and control, spurring calls for a greater scholarly focus on health literacy during the pandemic. Nevertheless, few studies have sought to qualitatively examine how individuals interpreted and assimilated health information at the initial wave of COVID-19 restrictions.

**Objective:**

We developed this qualitative study adopting chat-based focus group discussions to investigate how individuals interpreted COVID-19 health information during the first wave of COVID-19 restrictions.

**Methods:**

We conducted a qualitative study in Singapore to investigate how individuals perceive and interpret information that they receive on COVID-19. Data were generated through online focus group discussions conducted on the mobile messaging smartphone app WhatsApp. From March 28 to April 13, 2020, we held eight WhatsApp-based focus groups (N=60) with participants stratified by age groups, namely 21-30 years, 31-40 years, 41-50 years, and 51 years and above. Data were thematically analyzed.

**Results:**

A total of four types of COVID-19 health information were generated from the thematic analysis, labeled as formal health information, informal health information, suspicious health information, and fake health information, respectively. How participants interpreted these categories of information depended largely on the perceived trustworthiness of the information source as well as the perceived veracity of information. Both factors were instrumental in determining individuals’ perceptions, and their subsequent treatment and assimilation of COVID-19–related information.

**Conclusions:**

Both perceived trustworthiness of the information source and perceived veracity of information were instrumental concepts in determining one’s perception, and thus subsequent treatment and assimilation of such information for one’s knowledge of COVID-19 or the onward propagation to their social networks. These findings have implications for how policymakers and health authorities communicate with the public and deal with fake health information in the context of COVID-19.

## Introduction

### Background

The COVID-19 pandemic was declared a public health emergency of international concern by the World Health Organization (WHO) in January 2020 [1] and was subsequently declared a pandemic in March 2020 [2]. As of April 2022, globally, over 500 million people have been infected with the virus that causes COVID-19 and more than 6 million people have died from the disease [3]. Besides its impact on morbidity and mortality, COVID-19 has deeply impacted economies, health systems, and social lives globally [4].

The rapid spread of COVID-19 and its variants Delta and Omicron led to an urgent need for reliable, evidence-based, trustworthy, and updated health information that could help individuals to inform decision-making around COVID-19 prevention and management. Early dissemination of COVID-19 prevention guidelines by global and national authorities was accompanied by a concomitant rise of misinformation or fake news [5], termed an “infodemic” by the WHO Director-General Tedros Adhanom Ghebreyesus. The infodemic has caused concern for authorities due to its negative impacts on COVID-19 prevention and control [6], spurring calls for a greater scholarly focus on health literacy during the pandemic [7-9].

The infodemic has resulted in challenges in efficiently and trustworthily conveying reliable, rigorous COVID-19 information to the public. For example, a nationally representative online survey in Germany found that 47.8% of participants had trouble assessing if media information on COVID-19 could be trusted [10]. The infodemic has simultaneously facilitated the spread of conspiracy theories on COVID-19, including its origins and vaccines [11-13]. Studies also highlight how those with low levels of health literacy and those residing in low- to middle-income countries are at the greatest risk of succumbing to false or misleading pandemic-related information [10,14].

Past studies have attempted to determine the relationship between general literacy and COVID-19–related health literacy [15-17] or nuance the nature and types of COVID-19 information [18-20]. However, there has been minimal research to explore the factors that affect how individuals negotiate, construe, and interpret information in an infodemic. An understanding of such human factors, specifically how individuals interpret health information during emerging infectious disease contexts, is important for the widespread assimilation and integration of health information technologies in our ongoing management of the current pandemic and future pandemics.

### Use of Mobile Chat Apps for Focus Group Discussions

The use of focus group discussions (FGDs) for social science and health research, especially in reference to its methodological rigor for qualitative research, has been widely researched [21,22]. Such inquiries have extended to online FGDs, with their origins extending to the late 1990s with the use of emails and message boards [23], and subsequently with virtual discussion rooms and teleconferencing software [24,25].

Studies have found that online FGDs have important benefits relative to in-person groups. While responses were typically less detail-rich, researchers found that they were more immediate. Past studies also argued that online FGDs lack contextual clues that may inform perceived power differences between participants, which may facilitate the sharing of sensitive information and disagreements among participants [26,27]. Logistically, online groups can be more inclusive than traditional in-person FGDs, reducing physical access–related barriers to participation and allowing for participants to share diverse types of media throughout the course of the FGDs [28].

We also recognize the drawbacks of online FGDs compared to in-person groups. Online FGDs tend to generate lower word counts, shorter responses, and provide less detail or richness [25,26,29]. They also tend to have fewer group interactions and lower responsiveness to facilitators’ questions and probes [25,27,29].

Recent scholarship has highlighted the usefulness of mobile chat platforms such as WhatsApp in eliciting and generating qualitative data. Chen and Neo’s [30] study comparing indicators of data depth and breadth between WhatsApp-based and in-person FGDs in Singapore found that while data richness and detail in the WhatsApp FGDs did not match that of in-person groups, younger and more digitally savvy participants generated well-elaborated responses and were interactive within the chat groups. Findings from Colom’s [31] study in Western Kenya employing digital ethnographic methods, including the use of WhatsApp FGDs, also showed that the use of WhatsApp provided a high level of ecological validity [31].

### The COVID-19 Pandemic in Singapore

The first case of COVID-19 was reported in Singapore on January 23, 2020, prompting national authorities to implement a series of movement control measures to curb disease spread. One of the first steps taken involved the closure of entertainment establishments in late February 2020 following the change in Singapore’s Disease Outbreak Response System Condition (DORSCON) color code from yellow to orange. This signaled an official recognition of COVID-19’s severity and infectiousness, and a recognition that the disease had arrived in Singapore. A “circuit-breaker” period was implemented from April 7 to June 1, 2020, characterized by strict movement control measures aimed at curbing the growing incidence of COVID-19 cases in the community, and a “break the circuit” of transmission [32] period, including the closure of all nonessential services and a mask mandate. Since June 2020, Singapore has gradually eased these measures in phases; phase one took place between June 2 and June 18, 2020, which involved the progressive resumption of select businesses and activities.

### Study Rationale and Objective

Against the backdrop of information-related concern and policy responses in the early stages of the COVID-19 pandemic in Singapore, we developed this study to investigate how individuals interpreted COVID-19 health information during the first wave of COVID-19 restrictions.

## Methods

### Participants and Data Generation

We conducted a series of eight FGDs on WhatsApp between March 28 and April 13, 2020. Further details on how the FGDs were conducted have been published elsewhere [33]. We chose WhatsApp as our means of data collection because it is the most widely used mobile chat app in Singapore [34] and to protect the health and well-being of participants during the pandemic. Participants were recruited via an online flyer distributed on social media platforms, including Facebook, Twitter, and Instagram. Eligibility criteria were aged 21 years or older and being a Singapore citizen or permanent resident at the point of recruitment. Assuming varying levels of technological savvy and their impacts on group dynamics, participants were purposively recruited by strata according to the following age categories: 21-30 years, 31-40 years, 41-50 years, and 51 years and above. Within each group, we ensured a mix of participants of varying ethnicity, gender, and educational attainment. A summary of participant demographics for the study can be found in Table 1.

**Table 1:** Summary of participant demographics.

Variables			Participants, n
**Age (years)**			
	21-30	16	
	31-40	16	
	41-50	16	
	≥51	16	
**Gender identity**			
	Female	27	
	Male	20	
	Another gender	1	
**Race**			
	Chinese	42	
	Malay	2	
	Indian	2	
	Another race	2	
**Formal education attainment**			
	Secondary and below	1	
	Preuniversity	13	
	University	34	

### Design of FGDs

We conducted two FGDs per age strata, totaling eight FGDs with 6-8 participants per group and 60 participants overall. Each FGD was led by a main facilitator with two observers present. FGDs were conducted over 5 consecutive days, with new discussion topics introduced daily. Topics covered included knowledge and perceptions of COVID-19 and attitudes toward varying information sources.

We conducted both synchronous (ie, all participants were required to be online at a specific time for a specific duration) and asynchronous (ie, participants could reply at their convenience over the course of the day) sessions. Synchronous discussions of approximately 2 hours each were held on the first and last days of the study; asynchronous discussions were held on the second, third, and fourth days. For synchronous sessions, we prepared a list of questions that we had asked participants consecutively during the stipulated timing of 2 hours and participants were expected to stay online to participate at that time. For days that involved asynchronous participation, participants were told that questions would be posed from 9 AM all the way up until 6 PM that day, and that they could choose to answer at any point.

All chat transcripts, including media files such as photos, videos, and memes, were directly downloaded from the researchers’ WhatsApp mobile apps and stored in a secure, password-protected location accessible only to research team members. Participants were reimbursed SG $50 for their time (approximately US $35).

### Ethics Considerations

Ethics approval was obtained from the Saw Swee Hock School of Public Health Department Ethics Review Committee (SSHSPH-014). All participants provided documented informed consent before participating and completed a demographic questionnaire to indicate their interest to participate.

### Data Analysis

Data were analyzed by the lead author, adopting Braun and Clarke’s [35,36] six steps of reflexive thematic analysis. Both semantic and latent codes and themes were derived from the data without a pre-existing framework. Following the first two stages of familiarization and coding procedures in classic thematic analysis, the lead author noted clear patterns in how participants discussed and privileged varying forms of health information in the COVID-19 context. The lead author discussed these themes with coauthors to ensure that codes and developing themes were adequately fleshed out and authentic to the data generated [37]. Next, the lead author continued to code according to thematic analysis procedures, drawing links between various constructions and interpretations of health information and the factors underpinning them. At this stage, a typology of interpretation was developed by grouping types along two broad axes based on the perceived trustworthiness of an information source and perceived veracity of health information. All data were organized and analyzed using NVivo 11 software (QSR International Pty Ltd).

### Data Quality and Trustworthiness

We took several steps to improve the quality and trustworthiness of the data generated in this study. We worked to minimize biases around social desirability or discomfort with sharing by reiterating discussion ground rules on confidentiality, safety, respect, and voluntary participation on a daily basis. To reassure participants of our authenticity, we ensured that our WhatsApp profile pictures used a standardized template featuring a clear photo of our faces and our institutional affiliations. We also created separate “field notes” WhatsApp chat groups for the research team, which we used to reflect on group dynamics and themes in real time, which helped inform probes and prompts for more information and deepen our understanding of participants’ interpretations of health information.

## Results

### Overview

**Figure 1:**
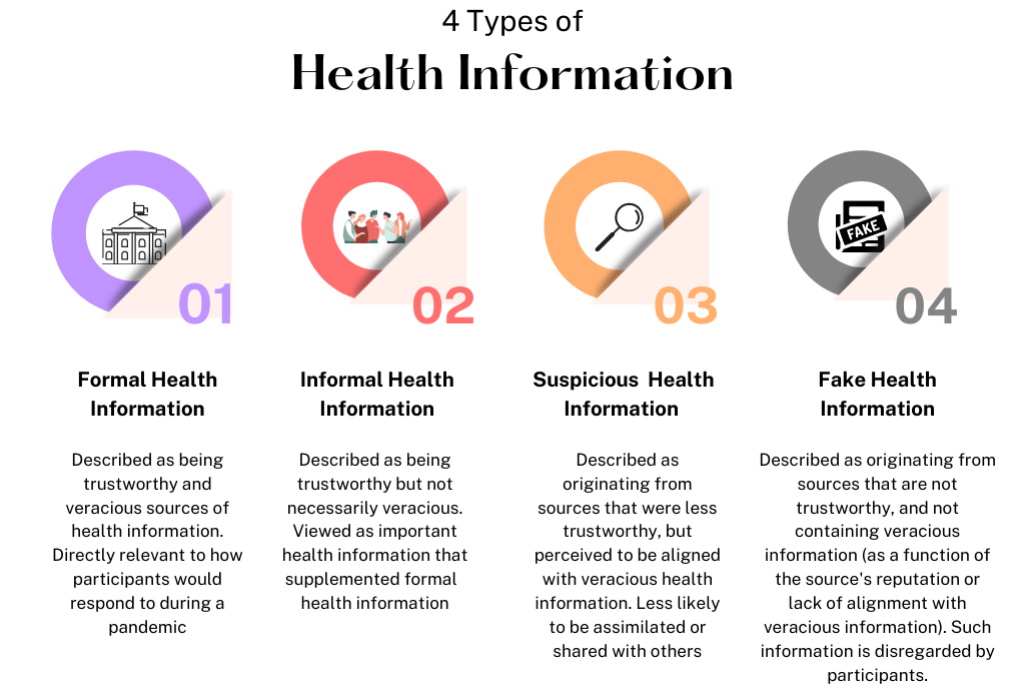
Summary of four types of health information interpreted by participants.

Based on our analysis of responses in our FGDs, we generated four types of health information, including *formal health information*, *informal health information*, *suspicious information,* and *fake health information*. These are summarized in Figure 1.

### Formal Health Information

Participants highlighted how they determined what was formal COVID-19 information through an understanding that such information was both trustworthy and veracious. Trustworthiness was premised largely on familiarity and past knowledge of an information source. Veracity was described by participants as being closely aligned with facts from “official” information sources. Participants described that formal health information around COVID-19 was directly relevant to how they would respond to the ongoing pandemic.

Participants described how they depended on trustworthy information sources such as local news channels and the national newspaper, The Straits Times, for formal COVID-19 information. Participants mentioned that the information source was considered trustworthy based on an understanding of its situation within a national regulatory information framework. This trustworthiness of an information source for formal COVID-19 information was reflected in participants’ beliefs that while some forms of reporting by the same news outlet may lack transparency due to censorship guidelines in Singapore, they could depend on said information source to report based on public interest. Participants also perceived such information to be accurate based on their understanding that it would be fact-checked. One participant described this as follows:

ST [The Straits Times] is a local news and it is under the supervision of IDA [Information Development Authority of Singapore], I know journalists check their facts and they are reviewed by an editor before it is published. […] The only thing is that there might be some info that is not shared for some reason (eg, not to alarm the public)

Other participants displayed slightly more critical attitudes toward the same information source but were able to distinguish between its position as a trustworthy information source for COVID-19 health information and its political intent. Similarly, they viewed the information source to be “factual and accurate” despite their preconceived notions of its role as state-controlled media:

Because it’s ST [The Straits Times], I trust that the article is factual and accurate, but sometimes I may be skeptical about the intent of ST articles. I guess there’s this general perception that ST is state-controlled media, so their articles may be biased towards the government or certain positions or promoting messages that are favorable to the government. But not that there’s anything very wrong about that. I will still read ST articles regardless. […] Yes, I read ST articles quite regularly, especially for local news like updates on the COVID-19 situation and related COVID-19 measures in Singapore.

Additionally, participants described how in the absence of such an understanding, they could fact-check on their own accord and rely on such information’s congruence with other information sources to determine veracity. Participants illustrated this in response to the facilitator’s prompt for participants’ sources of knowledge on the symptoms of COVID-19:

I first heard about it through credible news articles - but I’ve also googled to see what MOH [Ministry of Health] had to say. and I’ve checked the government resource (Singapore COVID-19 Symptom Checker [38])

Participant 1

From news media, but lately, just to prevent my drowning from all the news sites, I just follow CNA [Channel News Asia] and the government’s information.

Participant 2

### Informal Health Information

Participants described informal health information as originating from trustworthy sources, but may contain information that was not perceived to be veracious. Participants discussed how trustworthy information sources, including health information from traditional Chinese medicine practices, religious healing, or other informal sources of health information, served as important information that supplemented formal sources of health information. Participants reported relying on such forms of information as supplementary means of protecting oneself, rather than treating it as formal COVID-19 information.

Participants in the FGDs for older participants offered insight into how some information sources of health information were perceived to be trustworthy even if they did not provide veracious COVID-19 health information. When provided an image prompt depicting the use of onions, turmeric, and ginger to ward off COVID-19, participants discussed how health information from sources such as traditional Chinese medicine could be viewed as trustworthy, even though it was not perceived as being aligned with veracious information on COVID-19:

Yes, I heard onion is good even before this COVID-19. This has been circulating for a while already. I don’t believe it works this way but I can understand why it is being circulated. They are known to have anti-inflammatory properties when eaten so people might skew this info.

Participant 1

I believe they [Chinese people] know better than me. This is not supported by scientific research. […] Being elderly, more familiar with traditional Chinese medicine (TCM), but especially Chinese.

Participant 2

Other participants cited how friends served as trustworthy sources of information, even though they could not ascertain the veracity of such health information in the context of COVID-19 prevention. When prompted with the same picture from above, a participant from a separate FGD highlighted the following*:* “I heard onions do kill germs, and friends do practice it. But how effective, I don’t know.”

Other participants also discussed the use of a “silver ion spray”; one participant shared information about and promoted the product, citing its antiviral properties, even though such information was not specific to COVID-19:

Never seen or heard. But heard of silver ion spray. Please watch this video with a high-resolution camera showing how the virus can be spread. Don’t bother about the Japanese language. The images are enough to understand. [Video attachment of silver ion spray product]. It’s an alcohol-free antivirus spray that can kill up to 99.9% [of viruses]. It’s safe to be sprayed into the eye as well.

### Suspicious Health Information

Participants described suspicious health information as typically originating from less trustworthy information sources, even though it might be consistent with known, veracious COVID-19–related information. Such information was typically not viewed as a favorable information source that participants would assimilate or share with others. One participant pointed out how despite factual reporting of COVID-19 information from certain sources, they would “take it with a pinch of salt” due to perceived political biases of such publications:

We even discount [South China Morning Post] as credible. We need to analyze whether certain posts are politically motivated. News outlets like Fox News may be official and credible but I will take it with a pinch of salt.

When asked to discuss and compare three articles by various news outlets on the same topic, participants highlighted that their levels of trust in that same piece of health information varied depending on the source of the news article. One participant highlighted how it was difficult to measure trustworthiness among news articles and preferred to stick to government bodies as an authority for formal COVID-19 information. Participants also pointed out that a media outlet’s trustworthiness would also depend on the subject matter, and that they would trust varying sources depending on whether they were reporting on certain topics or geographies. A discussion by several participants highlights this nuance:

I’ll trust ST [The Straits Times] more because I've read articles from BBC [British Broadcasting Corporation] and SCMP [South China Morning Post], especially that latter, that have a very biased view against Singapore. Since ST is more towards the local context, as a Singaporean I’ll trust it more.

Participant 1

Depending on the locations that are being written about, I would trust different sources. For example, if I wish to find out about local updates, I would be inclined to read Straits Times instead.

Participant 2

### Fake Health Information

Participants described how fake health information usually came from untrustworthy sources and could be viewed as not being veracious through two mechanisms. First, it could be determined as a function of trustworthiness, in that information was not trustworthy owing to its historical and consistent lack of fact-checking. Therefore, information would be viewed as inaccurate regardless of its content. Second, information from such sources did not align or comport with veracious COVID-19 information propagated by official sources. In general, participants disregarded such forms of health information.

Broadly, participants across most FGDs described how forwarded WhatsApp messages from relatives were regarded as untrustworthy, and therefore contained unverified information on COVID-19. Participants from one FGD forwarded a text message to the group from a relative on the use of food to protect oneself from COVID-19, and subsequently commented on its trustworthiness:

I didn’t take it seriously because there was no trustworthy source cited.

Participant 1

Recently I couldn’t find vinegar in the supermarket. I asked my mummy’s circle [a motherhood-centered social group] why vinegar is OOS [out of stock]. According to them, some elderly believe drinking vinegar can protect themselves from COVID-19. Btw [by the way], I just need it for my gyoza [Japanese dumpling].

Participant 2

By contrast, participants in a separate FGD focused on COVID-19 prevention measures in their discussion on determining fake health information. One participant had forwarded a video that was circulating on WhatsApp of a police officer chatting with a civilian on the sidewalk next to a police van and issuing a fine. Another participant responded that it was untrustworthy by virtue of how it was shared (ie, through WhatsApp). However, while participants viewed forwarded messages as an untrustworthy source, they also described how they would discern if such information aligned with their knowledge of accurate and formal COVID-19 information. Specifically, the original poster approached this as *suspicious health information* instead and called this a “half-truth” due to the lack of trustworthiness of the source, although aligning with their understanding around current COVID-19 movement control measures:

[This video] Definitely, sent through WA [WhatsApp] groups.

Participant 1

I saw that too. I feel like it’s some sort of a half-truth, around people being issued notices [for flouting safe distancing measures]. I don’t know if the actual fines are legit [legitimate].

Participant 2

I think the fines are for people who do not observe safe distancing. The authorities encourage people to wear masks now even if well but it is not mandatory. So yeah, such forwarded message creates confusion.

Participant 1

## Discussion

### Principal Findings

Our study found that participants interpreted four types of health information, namely *formal health information*, *informal health information, suspicious health information,* and *fake health information*. These forms of health information were determined as a function of the perceived trustworthiness of a given information source and the perceived veracity of information. We discuss the implications of these findings in the context of existing studies on health literacy and approaches to dealing with the COVID-19 infodemic.

We found that participants were able to distinguish between formal and informal sources of health information. Determining if health information belonged to these respective categories would involve receiving information from trustworthy sources of information and assessing if it is veracious or not, which may be a given for certain sources of formal information or requires further fact-checking. For study participants, supplementary health information sources played an important role in providing additional perceived protections from COVID-19, although participants recognized that such information was not official or they were unaware of the mechanisms through which it would positively impact COVID-19 prevention efforts. This was especially salient for the older age groups, who discussed how certain forms of trustworthy sources, including alternative healing, traditional Chinese medicine, or frameworks of cultural beliefs, provided resources to supplement their COVID-19 prevention behaviors. Studies in other settings have found that complementary, traditional, or alternative forms of medicines have played a strong role in the alleged treatment and prevention of COVID-19 [39-41], despite the lack of rigorous randomized controlled trials to underpin such evidence [42,43].

The trustworthiness of a source played a key role in determining if participants would assimilate or share certain forms of information. In general, both suspicious and fake health information led to participants having reservations around assimilating and sharing such information, even if they knew that the information had some “truth” in it due to existing knowledge of formal and accurate COVID-19 information. Past studies show that trust in a particular source plays a strong role in eventual health information–seeking behaviors [44,45]. In turn, those with higher levels of health literacy tended to trust information from health care professionals and were less likely to do so for information from social media, celebrities, and friends [46]. This comports with our finding that older adults, who have been found in multiple settings to have limited health literacy [47-49], may also rely on complementary or alternative sources of health information to inform their COVID-19 prevention efforts. Several scholars have also reviewed the antecedents of trust for online health information [50,51], while others have proposed sociodemographic correlates of trust for varying sources of offline health information [52,53].

Overall, our findings indicate that individuals may label the same piece of health information differently depending on their perceptions of a source’s trustworthiness and veracity of health information. This finding aligns with research that points out a shift toward a “posttruth” era, one that situates truthful information as at risk of being undermined by “alternative facts” or misinformation [54]. The findings of this study are also consistent with past scholarship that posits how the dynamics of belief formation and the definition of truth are being contested in contemporary societies [55], which may impact health and media literacy in society.

### Strengths and Limitations

We identified three key study strengths. First, we generated a dynamic framework to understand how people approach or interpret health information in the context of an infodemic. The typology provides opportunities for policymakers to propose interventions and communication strategies that may enable effective and rapid communication in a pandemic, while acknowledging the varied ways in which individuals interpret information. Second, WhatsApp proved to be an appropriate platform for discussions on health literacy in times of an infodemic, as participants were able to share and forward information and media that helped initiate dynamic discussions on their approaches to determining the trustworthiness and/or veracity of such information. Third, WhatsApp-based FGDs allowed us to continue with the study despite the ongoing movement control restrictions that were progressively implemented midway through the study, thus allowing us to retain the integrity of our proposed methods across all groups while keeping our participants safe.

We are also mindful of several limitations. First, the use of WhatsApp FGDs did not allow facilitators to make sense of nonverbal cues such as the use of body language, which might have diminished rapport between participants and researchers. Furthermore, the lack of tone that would normally be present in verbal communication meant that some meanings could have been misconstrued by the facilitators. Nevertheless, our team members were able to analyze emojis, images, or stickers to gain more context in lieu of such cues. These provided additional context for tone when analyzing participants’ responses:

This is the most prolonged WhatsApp conversation I’ve been a part of 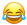 […]

Participant 1

Thank you for the great insights! Have a great week ahead 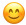 […]

Participant 2

Not that I remember 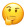

Participant 3

Furthermore, having experience within the team in employing this method, steps were taken to mitigate such methodological shortcomings, as articulated in the Methods section [33].

### Conclusion

We conclude with two specific recommendations for health authorities and policymakers to enhance effective communications during a health infodemic such as during the COVID-19 pandemic. These recommendations aim to intervene on levels of perceived trust in health information sources and the perceived veracity of health information. In the context of the trustworthiness of health information sources, we recommend policies that shape norms and build trust in select sources of information to combat a glut of health information. The nature of trust and trustworthiness of sources is complex, and draws on participants’ past experiences and literacy around a particular source’s attributes and authority. Scholarly work on the antecedents of trust for online health information might serve as useful starting points for interventions aimed at promoting trust in key sources of information. Such efforts should be implemented well before times of crises and infodemics.

Second, we recommend acknowledging nuances between formal, evidence-based information and information on alternative or complementary medicine or treatments, and implementing an information framework that distinguishes between, yet supports both. Our results suggest that the recognition of supplementary health information may not necessarily be harmful, given that such information may be used to complement formal health information in times of a pandemic. However, this presupposes a strong understanding of what comprises formal and veracious health information and the information architecture that supports such interpretations. Upon establishing key sources of veracious information, individuals may be better equipped to distinguish what *must* be done to protect themselves in a pandemic, relative to what *can* be done as a means of supplementing formal COVID-19 prevention measures.

## Abbreviations

DORSCON: Disease Outbreak Response System Condition
FGD: focus group discussion
WHO: World Health Organization
